# Relationship between ultrasonographic findings and subscales of the Knee Injury and Osteoarthritis Outcome Score in patients with early knee osteoarthritis: a multicenter study

**DOI:** 10.1007/s10396-024-01498-w

**Published:** 2024-09-25

**Authors:** Yushin Mizuno, Yasushi Takata, Yosuke Shima, Kenichi Goshima, Kazunari Kuroda, Tomoyuki Kanayama, Yoshihiro Ishida, Naoki Takemoto, Manase Nishimura, Takuya Sengoku, Satoru Demura, Junsuke Nakase

**Affiliations:** 1 Department of Orthopaedic Surgery, Graduate School of Medical Sciences, Kanazawa University, 13-1 Takaramachi, Kanazawa, Ishikawa 920-8641 Japan; 2 Section of Rehabilitation, Kanazawa University Hospital, Kanazawa, Ishikawa Japan; 3 Department of Orthopaedic Surgery, KKR Hokuriku Hospital, Kanazawa, Ishikawa Japan; 4 Department of Orthopaedic Surgery and Joint Reconstructive Surgery, Kanazawa Munehiro Hospital, Kanazawa, Ishikawa Japan; 5 Department of Orthopaedic Surgery, Yawata Medical Center, Komatsu, Ishikawa Japan

**Keywords:** Correlation, Early knee osteoarthritis, Knee Injury and Osteoarthritis Outcome Score, Multicenter study, Ultrasonographic findings

## Abstract

**Purpose:**

To characterize the ultrasonographic findings of patients with early knee osteoarthritis (KOA) and determine which findings were associated with the Knee Injury and Osteoarthritis Outcome Score (KOOS) subscale.

**Methods:**

The study included 98 knees (35 men, 63 women, 60.3 ± 11.5 years) diagnosed with early KOA with no major deformity radiographically, but with pain during activity and tenderness in the medial knee. Synovial hyperplasia in the suprapatellar bursa, knee joint effusion, horizontal tear of the medial meniscus (MM), osteophytes of the medial condyle of the femur and tibia, blood flow signals in the synovium of the suprapatellar bursa, medial collateral ligament bursa, infrapatellar fat pad, MM extrusion (MME) in the supine and upright positions, and the amount of change in MME were observed using ultrasonography.

**Results:**

Correlations (*p* < 0.05) were found between the presence of synovial hyperplasia of the suprapatellar bursa (*r*<-0.20) and amount of MME in the upright position (*r*< − 0.24) and all KOOS subscales. Presence of joint effusion and the four KOOS subscales except quality of life (QOL) were correlated (*p* < 0.05). Partial correlation coefficients showed correlations (*p* < 0.05) between knee joint effusion and symptoms (*r* = 0.299) and activities of daily living (ADL) (*r* = 0.254) of the KOOS subscales, and between MME in the upright position and symptoms (*r*= − 0.263), pain (*r*= − 0.256), and ADL (*r*= − 0.212).

**Conclusion:**

Quality and difficulty of life of patients with early KOA may be influenced by synovial hyperplasia in the suprapatellar bursa, joint effusion, and MME values in the upright position. Among them, knee joint effusion and amount of MME in the upright position were independently associated with the KOOS subscales.

## Introduction

Knee osteoarthritis (KOA) is a disease that significantly reduces activities of daily living (ADL) and quality of life (QOL), and the number of patients with KOA is increasing globally [1]. In addition, pain, functional decline, and medical costs associated with KOA pose a burden to society [2, 3]. In Japan, a country of longevity, the number of patients with KOA has exceeded 8 million for the past 15 years [4]. Several treatment approaches for KOA have been investigated for symptom relief [5]; however, the pathophysiology of KOA is complex, and there is no consensus on the disease progression from onset to severe disease. The severity of KOA implies the need for surgical treatment, such as total knee arthroplasty [6]; therefore, early intervention is desirable and has attracted scientific attention in recent years [7–9].

Therefore, the new concept of “early KOA” was proposed to aid early diagnosis [9, 10]. However, the diagnostic criteria for early KOA are still under debate, and the definition of it remains ambiguous [11]. At this stage, the common denominator is the presence of knee pain but little or no deformity with a Kellgren–Lawrence (K–L) grade of 0 or 1 on radiographic images of the tibiofemoral joint [9–12]. This means that the knee joint components, such as the cartilage, subchondral bone, and meniscus, undergo structural changes before abnormal findings become visible on radiographic images. Magnetic resonance imaging (MRI) detects these observations [9] and has been suggested to be effective in detecting early KOA [13, 14]. However, MRI is not included in the diagnostic criteria for early KOA because of the large installation cost of MRI, high cost of healthcare for patients, and scarce MRI equipment itself in many countries [10, 15]. In contrast, ultrasonography is cheaper than MRI and allows repeated measurements with ease [16]. Hence, the mainstay of diagnosis and treatment of early KOA could be ultrasonography based on these advantages, leading to improved observation of the knee joint components.

The prevalence of early KOA is 9.5% in males and 15.0% in females, with a higher prevalence in middle-aged females in Japan [17]. However, ultrasonographic findings characteristic of these patients with early KOA have not been elucidated to date. It is also not known how these ultrasonographic findings are associated with the patients’ problems in daily life. These data may help to identify some of the complex pathophysiology of early KOA and may aid in prevention of KOA progression and symptom improvement. Therefore, we aimed to characterize ultrasonographic findings in patients with early KOA with medial knee pain and tenderness and a K–L grade of 1 or less, and determine which ultrasonographic findings were associated with the KOOS subscales. Based on the results of similar previous studies in patients with KOA and patients with early KOA [18–21], we hypothesized that the synovial hyperplasia in the suprapatellar bursa, knee joint effusion, presence of osteophytes, and amount of medial meniscus extrusion (MME) would be related to the patients’ difficulties in daily living as expressed by KOOS.

## Materials and methods

This cross-sectional study was conducted after obtaining approval from our institution’s Medical Ethics Review Committee (Approval number: 2022-12 [714071]) and collecting patients’ information from several hospitals. Written informed consent was obtained from all participants for the collection of the information.

### Participant recruitment

**Fig. 1:**
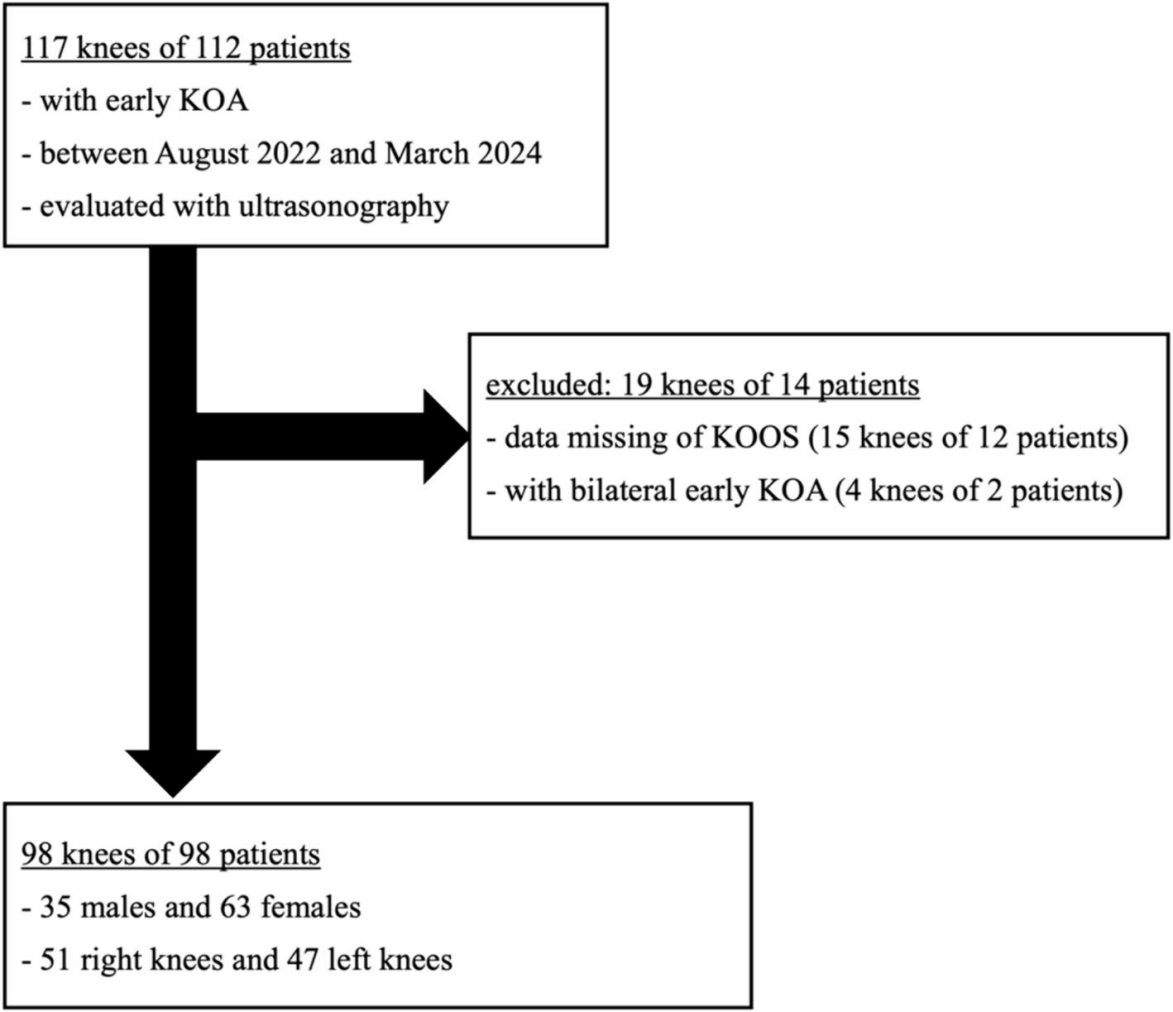
Participant recruitment. This study was conducted from August 2022 to March 2024 in patients with knee pain without significant deformity on radiographic images. Of these, 14 patients (19 knees) were excluded because of missing data, and 98 patients (98 knees) were included in this study.

We examined 117 knees of 112 patients with early KOA with medial knee pain during activity, tenderness, and K-L grade ≤ 1 of the tibiofemoral joint who presented to our or affiliated hospitals between August 2022 and March 2024 using ultrasonography. The diagnosis of early KOA was made by seven orthopedic surgeons with at least 5 years of experience and familiarity with knee treatment. Patients with knee pain due to trauma or inflammatory disease or with postoperative knees were not included in the study. None of these early KOA patients had obvious osteoarthritis of the patellofemoral joint. In addition, patients with missing KOOS results required for the study and with bilateral knees diagnosed with early KOA were excluded. The data for 98 knees of 98 patients (35 males and 63 females, 60.3 ± 11.5 years) were ultimately analyzed (Fig. 1).

### Ultrasonographic evaluation

SONIMAGE HS1 (KONICA MINOLTA JAPAN, Inc., Tokyo, Japan) or Venue Go (GE Healthcare Japan, Tokyo, Japan) with a linear transducer (18–4 MHz) was used as the ultrasonography system. Ultrasonography was performed by seven orthopedic surgeons with at least 5 years of clinical experience and familiarity with ultrasonography-based knee treatment. The B-mode and Doppler settings of each device were adjusted by the operator so that each region of interest could be clearly identified. No changes were made to the settings except for the size, depth, and focus of the images and the size and position of the color box for the Doppler mode.

In this study, the following 11 parameters were examined using ultrasonography: synovial hyperplasia in the suprapatellar bursa, knee joint effusion, horizontal tear of the medial meniscus (MM), osteophytes of the medial condyle of the femur, osteophytes of the medial condyle of the tibia, blood flow signals in the synovium of the suprapatellar bursa, blood flow signals in the medial collateral ligament (MCL) bursa, blood flow signals in the infrapatellar fat pad, MME in the supine position, MME in the upright position, and amount of change in MME (ΔMME) in the patients’ knees. These investigations are commonly performed by clinicians in daily practice for patients with knee osteoarthritis, and these checks were carried out on the patient’s first visit to our institution.

**Fig. 2:**
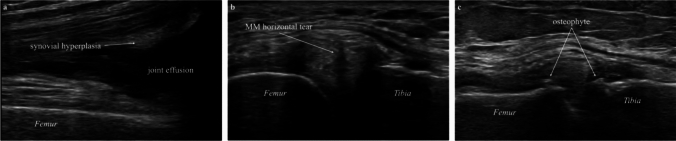
Ultrasonographic findings of synovial hyperplasia, joint effusion, horizontal tear of the medial meniscus, and osteophytes of the medial condyle of the femur and tibia Synovial hyperplasia and joint effusion were observed in the suprapatellar bursa (**a**). The largest portion of the synovial thickened area and the joint effusion was measured, and a diameter ≥4 mm was considered positive. Horizontal tears of the medial meniscus and osteophytes were observed on the long-axis image, in which the medial collateral ligament was most clearly depicted. A horizontal tear of the medial meniscus was considered positive if there was a hypoechoic area in the meniscal parenchyma (**b**). Osteophytes were identified bilaterally on the femoral and tibial sides. In the presence of osteophytes, bony prominences were identified at the site of the articular cleft, and in the case of cartilaginous osteophytes, a double overlap of hyperechoic areas was identified (**c**)

Five parameters (synovial hyperplasia in the suprapatellar bursa, knee joint effusion, horizontal tear of the MM, osteophytes of the medial condyle of the femur, and osteophytes of the medial condyle of the tibia) were assessed positively and negatively. Synovial hyperplasia and knee joint effusion were determined using long-axis images of the suprapatellar bursa during slight knee flexion [22]. The longest diameter of the synovial thickened area and the joint effusion was measured, and a value ≥ 4 mm was considered positive (Fig. 2a) [18, 19, 23]. Horizontal tears of the MM and osteophytes of the medial condyle of the femur and tibia were assessed medial to the knee joint. The transducer was placed proximal to the medial femoral epicondyle as a marker, parallel to the MCL, and where the MCL was significantly delineated to ensure reproducibility. Consequently, the patients’ knees were extended, and long-axis images were acquired. Horizontal tear of the MM was considered positive if there was hypoechogenicity within the meniscus (Fig. 2b) [24, 25]. The medial knee joint cleft was observed for osteophytes, and the presence of osteophytes (or cartilage osteophytes) in the same area was considered positive (Fig. 2c) [26, 27].

**Fig. 3:**
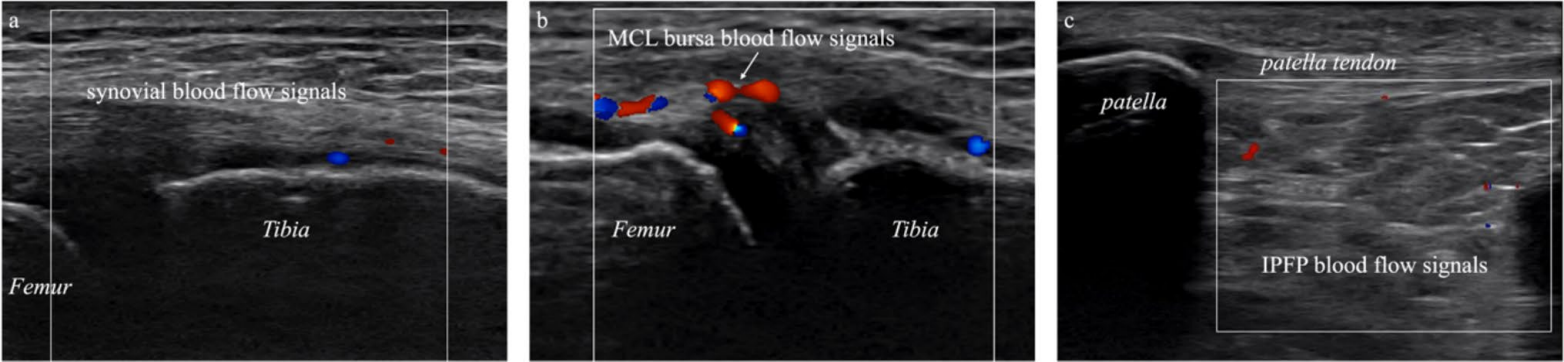
Ultrasonographic findings of synovial, medial collateral ligament bursa, and infrapatellar fat pad blood flow signals The degree of increased blood flow was evaluated in the synovium of the suprapatellar bursa (**a**), medial collateral ligament bursa (**b**), and infrapatellar fat pad (**c**). In accordance with previous studies, the degree of the blood flow signal was classified into four levels (absent, mild, moderate, and marked or severe)

The blood flow signals in the three knee regions were classified into four levels, i.e., absent, mild, moderate, marked/severe [28], according to their degree, using the Doppler mode of the ultrasonography equipment. Increased synovial blood flow signals (Fig. 3a) were observed on long-axis images in the same areas where synovial hyperplasia and joint effusion were observed [19]. The MCL bursa is located between the superficial and deep layers of the MCL [29] and can be identified using ultrasonography [30]. The increased blood flow signal in the MCL bursa (Fig. 3b) was determined at this location with long-axis images. Observation of the blood flow signal in the infrapatellar fat pad (Fig. 3c) was assessed using long-axis images at the position of the patellar tendon with the knee joint in mild flexion [31, 32].

**Fig. 4:**
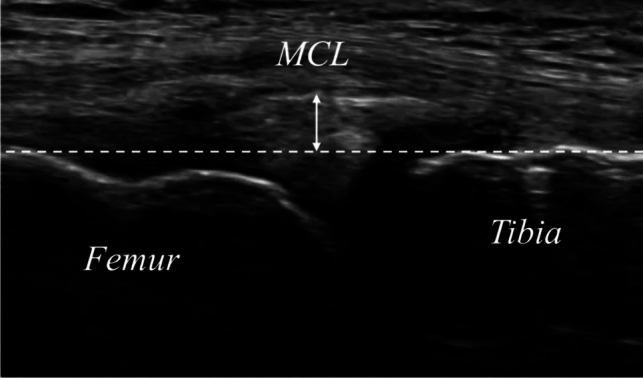
Ultrasonographic findings of medial meniscus extrusion Medial meniscal extrusion (MME) was evaluated on a longitudinal image that significantly depicted the medial collateral ligament (MCL). MME is a straight line parallel to the MCL passing through the outer margin of the tibia. MME was defined as the amount of perpendicular deviation from a straight line parallel to the MCL passing through the outer tibial border. In this study, MME was measured in both the supine and upright positions, with the difference defined as ΔMME (upright - supine)

MME was measured under two conditions: supine and upright [33]. MME was defined as the displacement from the margin of the tibial plateau and was measured as the distance (in mm) between the margin of the tibial plateau and the peripheral border of the meniscal body (Fig. 4) [34]. For measurements in the supine position, the patients’ knees were kept in extension. Measurements in the upright position were also performed in the same manner, with the patients instructed to hold both lower limbs shoulder-width apart [33]. The MME was calculated by subtracting the MME value in the supine position from that in the upright position. The reproducibility of MME measurements in the supine and upright positions using ultrasonography has been confirmed in previous studies [33, 35].

### Patient-oriented assessment with KOOS

The patients were asked to complete the Japanese KOOS (J-KOOS) during the waiting period of their first visit to our institution. The timing of this questionnaire was equivalent to that of the ultrasonographic examination. There was no intervention by the medical staff when patients filled in the questionnaire; only the patient’s intentions were included in its content. The KOOS has five subscales and is one of the patient-reported outcome measures for patients with any knee disease, allowing for the observation of the effects of several diseases [36, 37]. Similar to the original version, the J-KOOS consists of 42 questions categorized into five subscales: symptoms, pain, ADL, sports, and QOL, and its validity was confirmed by Nakamura et al. in 2011 [38]. Grading was also carried out according to the original method [36]. Each question was scored on a five-point scale (in 25 increments), with 0 and 100 as the worst and best conditions, respectively; the average of all questions in each subscale was used as the score for that subscale. Therefore, the minimum and maximum scores were 0 and 100, respectively, for each subscale.

### Statistical analyses

First, positive rates (%) were calculated to characterize the ultrasonographic findings in patients with early KOA. For parameters with a 2-grade scale, the positive rate was calculated as positive/98, and for parameters with a 4-grade scale, the positive rate was calculated in the same way, with all but absent being considered positive.

The IBM SPSS Statistics for Mac version 29.0.1.0 (IBM Corp. Armonk, NY, USA) was used for all the statistical analyses. As a preliminary step to clarify the relationship between the various ultrasonographic findings and the KOOS subscale, the normality of continuous variables was confirmed using the Shapiro-Wilk test. Pearson’s correlation coefficient was calculated for data in which normality was found in both parameters revealing the relationship, and Spearman’s rank correlation coefficient was calculated for the rest. The partial correlation coefficients were also calculated to confirm the independence of findings that were significantly associated with KOOS subscales. Statistical significance was set at *p* < 0.05.

To measure the statistical power of the results, a post-hoc test using G*Power 3.1 (Franz Paul, Kiel, Germany) was performed [39]. The input parameters were two-tailed, the significance level α was 0.05, and the total sample size was 98.

## Results

Table 1 presents the details of the 98 knees of the 98 Japanese patients included in the study.

**Table 1:** Patients’ information

Parameter	
Sex (patients; male and female)	35 and 63
Age (years; mean ± SD)	60.3 ± 11.5
Height (cm; mean ± SD)	161.8 ± 9.4
Weight (kg; mean ± SD)	62.7 ± 13.2
Body mass index (kg/m^2^; mean ± SD)	24.1 ± 5.7
Involved side (knee; right and left)	51 and 47

*SD* standard deviation

The percentages of patients with early KOA with synovial hyperplasia identified in the suprapatellar bursa, joint effusion, horizontal MM tears, osteophytes of the medial condyle of the femur, and osteophytes of the medial condyle of the tibia observed medially in the knee were 9.2%, 38.8%, 60.2%, 48.0%, and 61.2%, respectively. In addition, 30.6%, 45.9%, and 24.5% of the patients had increased mild blood flow signals associated with synovial hyperplasia, MCL bursa, and infrapatellar fat pad, respectively. The mean MME was 2.69 mm in the supine position and 3.12 mm in the upright position. This showed that the MME of patients with early OA increased by approximately 0.4 mm when they were in the upright position (Table 2).

**Table 2:** Summary of ultrasonographic findings in patients with early knee osteoarthritis

Parameter		Positive rate
Synovial hyperplasia in the suprapatellar bursa (negative and positive)	89 and 9	9.2%
Knee joint effusion (negative and positive)	60 and 38	38.8%
Horizontal tear of the medial meniscus (negative and positive)	39 and 59	60.2%
Osteophyte of the medial condyle of femur (negative and positive)	51 and 47	48.0%
Osteophyte of the medial condyle of tibia (negative and positive)	38 and 60	61.2%
Blood flow signals in the synovium of the suprapatellar bursa (absent, mild, moderate, marked or severe; positive: mild, moderate, marked or severe)	68, 21, 7, or 2	30.6%
Blood flow signals in the MCL bursa (absent, mild, moderate, and marked or severe; positive: mild, moderate, and marked or severe)	53, 23, 14, or 8	45.9%
Blood flow signals in the infrapatellar fat pad (absent, mild, moderate, marked or severe; positive: mild, moderate, marked or severe)	74, 18, 5, or 1	24.5%
MME in supine position (mm; mean ± SD)	2.69 ± 1.19	–
MME in upright position (mm; mean ± SD)	3.12 ± 1.23	–
Amount of change in MME (mm; mean ± SD)	0.43 ± 0.85	–

*MCL* medial collateral ligament, *MME* medial meniscus extrusion, *SD* standard deviation

The mean values of the KOOS subscales of symptoms, pain, ADL, sports, and QOL were 65.0, 59.2, 73.5, 44.3, and 39.7, and the median values of them were 67.9, 58.3, 75.8, 40.0, and 37.5, respectively (Table 3).

**Table 3:** Summary of Knee Injury and Osteoarthritis Outcome Scores (KOOS) in patients with early knee osteoarthritis

Parameter	Mean ± SD (points)	Median	Minimum value	Maximum value
Symptoms	65.0 ± 19.3	67.9	14.3	100
Pain	59.2 ± 18.5	58.3	8.3	94.4
ADL	73.5 ± 17.0	75.8	27.9	100
Sports	44.3 ± 26.0	40.0	0	100
QOL	39.7 ± 22.3	37.5	6.3	100

*SD* standard deviation, *ADL* activities of daily living, *QOL* quality of life

Normality of continuous variables was observed for MME in upright position, symptoms and pain of the KOOS subscales. As for the primary outcome of this study, correlations (*p* < 0.050) were seen between the presence of synovial hyperplasia in the suprapatellar bursa (*r*<−0.200) and the amount of MME in the upright position (*r*<−0.240) and all five KOOS subscales (Table 4). A significant correlation (*p* < 0.050) was found between the presence of joint effusion and the four KOOS subscales other than QOL (Table 4). Furthermore, partial correlation coefficients for these parameters with each other as control variables showed no significant relationship between synovial hyperplasia in the suprapatellar bursa and any of the KOOS subscales (*p* > 0.050). However, significant correlations (*p* < 0.050) were found between knee joint effusion and symptoms (*r* = 0.299) and ADL (*r* = 0.254) of the KOOS subscales, and between MME in the upright position and symptoms (*r*=−0.263), pain (*r*=−0.256), and ADL (*r*=−0.212) (Table 5).

**Table 4:** Correlation between ultrasonographic findings and Knee Injury and Osteoarthritis Outcome Score (KOOS) subscales in patients with early knee osteoarthritis

	Symptoms	Pain	ADL	Sports	QOL
Synovial hyperplasia in the suprapatellar bursa	− 0.224*	− 0.230*	− 0.292**	− 0.290**	− 0.208*
Knee joint effusion	− 0.366**	− 0.233*	− 0.309**	− 0.213*	− 0.194
Horizontal tear of the medial meniscus	− 0.031	0.022	− 0.045	− 0.003	0.015
Osteophyte of the medial condyle of femur	− 0.104	− 0.159	− 0.036	− 0.050	− 0.092
Osteophyte of the medial condyle of tibia	− 0.002	0.112	0.159	0.082	0.051
Blood flow signals in the synovium of the suprapatellar bursa	− 0.142	− 0.138	− 0.132	− 0.102	− 0.098
Blood flow signals in the MCL bursa	0.018	− 0.166	− 0.044	0.014	0.049
Blood flow signals in the infrapatellar fat pad	0.006	− 0.001	0.026	0.081	0.107
MME in supine position	− 0.140	− 0.194	− 0.197	− 0.161	− 0.188
MME in upright position	− 0.339**	− 0.334**	− 0.270**	−0.246*	− 0.263**
Amount of change in MME	− 0.156	− 0.121	− 0.069	− 0.085	− 0.084

All the values in the table indicate correlation coefficients

*ADL* activities of daily living, *QOL* quality of life, *MCL* medial collateral ligament, *MME* medial meniscus extrusion

*p< 0.050

**p< 0.010

**Table 5:** Partial correlation coefficients for parameters with significant correlations between ultrasonographic findings and Knee Injury and Osteoarthritis Outcome Score (KOOS) subscales in patients with early knee osteoarthritis

	Control variables	Symptoms	Pain	ADL	Sports	QOL
Synovial hyperplasia in suprapatellar bursa	Knee joint effusion and MME in upright position	0.013	0.098	0.143	0.178	0.095
Knee joint effusion	Synovial hyperplasia in suprapatellar bursa and MME in upright position	0.299**	0.146	0.254*	0.103	0.096
MME in upright position	Synovial hyperplasia in suprapatellar bursa and knee joint effusion	− 0.263*	− 0.256*	− 0.212*	− 0.158	− 0.184

All the values in the table indicate correlation coefficients

*ADL* activities of daily living, *QOL* quality of life, *MME* medial meniscus extrusion

*p< 0.050

**p< 0.010

The power scores of the obtained results were 0.54 and 0.82 for an effect size of 0.205 and 0.3, respectively.

## Discussion

We characterized the ultrasonographic findings and KOOS subscales in patients with early KOA. We observed that synovial hyperplasia of the suprapatellar bursa, joint effusion, and MME in the upright position were associated with difficulties in daily life in patients with early KOA. Among them, knee joint effusion was significantly related to symptoms and ADL among the KOOS subscales, and MME in the upright position was also significantly related to symptoms, pain, and ADL in that subscale independently. However, all significant correlations have been considered as weak correlations.

The characteristics of ultrasonographic findings in patients with early KOA have not been well reported. In a study by Abbasi et al. [18] in patients with KOA (including early KOA) with knee pain, synovitis (synovial hyperplasia > 4 mm) was observed in approximately one-quarter of patients and joint effusion (> 4 mm) in approximately half. In contrast, in early KOA patients in our study, synovial hyperplasia was rarely observed, but joint effusion was found in approximately one-third of the patients. The difference in these findings may partially be because patients with early KOA have a shorter disease duration than patients with advanced KOA, and this may reflect part of the pathophysiology of early KOA. In addition, these authors also noted that synovitis and joint effusion were associated with Western Ontario and McMaster Universities Osteoarthritis Index (WOMAC) pain, physical function, and overall subscore on ultrasonographic evaluation. The finding that the presence of synovial hyperplasia and joint effusion were associated with the majority of WOMAC scores is similar to the results obtained in our study using the KOOS and is of equal significance as a certain relationship between WOMAC and KOOS scores in patients with KOA has been reported [40]. In addition, this correlation is also significant as it was also observed in a study involving approximately 4,000 Chinese individuals living in the same Asian region as the Japanese that revealed that synovial hyperplasia and joint effusion were associated with pain assessment using the WOMAC [19]. However, these studies did not evaluate the independence of ultrasonographic findings on the WOMAC score. In our study, we went one step further and found that in patients with early KOA, synovial hyperplasia in the suprapatellar bursa on ultrasonographic examination was not independent of the KOOS subscales. This suggests that synovial hyperplasia in the suprapatellar bursa is closely related to other factors and is not independently associated with the KOOS subscales.

Regarding synovitis (increased blood flow signals in the synovium) detectable with the Doppler mode, a study of 453 knees in patients with KOA showed that moderate or severe synovitis was strongly associated with knee joint symptoms, including pain [41]. However, our study showed no significant correlation between increased synovial blood flow signals and KOOS subscale scores. This may be because of the relatively mild synovitis in the patients with early KOA. We observed that only nine (9.2%) of the 98 knees had moderate or severe synovitis. A mild degree of synovitis may be a characteristic of patients with early KOA.

The amount of MME in the supine position was not associated with any of the KOOS subscales, whereas the amount of MME in the upright position was related to all subscales, and the amount of MME in the upright position was independently related to symptoms, pain, and ADL among the KOOS subscales. Previous reports have revealed an association between the amount of MME in the supine position and knee symptoms in patients with KOA [20, 21]. Our results are contrary to these findings. In addition, the previous studies did not examine the relationship between the amount of MME in the upright position and knee symptoms. Our study, to our knowledge, is the first study that revealed that the amount of MME in the upright position in patients with early KOA is negatively correlated with the KOOS subscales, with a correlation coefficient of about − 0.300. The difference in the results between the supine and upright positions implies that the amount of MME in the upright position is relatively related to knee symptoms in patients with early KOA. These results may also depend on the fact that most of the KOOS subscales assess symptoms during upright movements. Furthermore, ultrasonography allows immediate and easy measurement of MME in the upright position, and significant positive correlations with MME measured using MRI have also been observed [35]. Therefore, MME evaluation in the upright position may be useful for accurately identifying subjective knee symptoms in patients with early KOA.

We did not observe a statistical association between osteophytes of the medial condyle of the femur and tibia observed in the medial knee joint and the KOOS subscales. In contrast, several studies have reported an association between pain and osteophytes of the medial condyle of the femur and tibia, as evaluated using ultrasonography [20, 21]. This difference in the results may be related to the different methods used to evaluate the osteophytes. In previous studies, the degree and actual size of osteophytes were evaluated numerically. In contrast, we evaluated the presence and absence of osteophytes. Additionally, we evaluated only the correlation between the presence of osteophytes and KOOS subscale scores. Therefore, the relationship between osteophytes and knee symptoms is not completely ruled out, suggesting that there are some associations that cannot be detected by presence or absence alone.

In summary, we identified the characteristics of ultrasonographic findings and KOOS subscales and their relationships in patients with early KOA. However, significant correlations were weak. Furthermore, the causes of knee symptoms, including pain, vary widely [18, 20, 21, 41–46], and it is unlikely that only one ultrasonographic finding has a significant impact on the KOOS subscales. The weak correlation coefficients found in this study suggests that the relationship between ultrasonographic findings and difficulties in daily living in patients with early KOA is associated with complex factors that were not examined in this study. However, the finding that knee joint effusion and MME in the upright position have an independent relationship with some of the KOOS subscales would suggest that these two ultrasonographic findings may have some significance in the difficulty of life of patients with early KOA.

Our study had a few limitations. First, the ultrasonographic findings may be poorly reproducible, a disadvantage of a multicenter study. However, the reproducibility of these findings is reportedly highly reliable in previous studies [33, 35] and may not have significantly impacted our study results. Second, the participants included in this study were all Japanese, and our results may not apply to other races with different basic skeletons. However, our results may be particularly useful in Japan, a country with a long life expectancy. There was also variation in the period between the perception of knee pain and the ultrasonographic examination or KOOS subscale interview in the patients included in the study. However, some patients may not visit a hospital immediately after becoming aware of knee pain, making standardization difficult. In addition, a lack of data from the same generation of healthy participants without knee pain precludes discussion of how the data obtained in this study differ from normal. Therefore, additional studies in healthy subjects of the same age group will further clarify the characteristics of patients with early KOA. Finally, the causal relationship between ultrasonographic findings and the KOOS subscales is unknown because we only observed correlations. To date, the details of early KOA, including its pathogenesis, have not been elucidated. However, our study results may contribute to further understanding of the pathogenesis of early KOA. Further scientific investigations will reveal a causal relationship between these findings and the pathogenesis of early KOA.

## Conclusion

KOOS subscale scores were significantly associated with synovial hyperplasia of the suprapatellar bursa, joint effusion, and amount of MME in the upright position. Among them, synovial hyperplasia of the suprapatellar bursa and the amount of MME in the upright position were independently associated with the KOOS subscales. However, these relationships were weak, which suggests that the relationship between ultrasonographic findings and difficulties in daily living in patients with early KOA is associated with complex factors that were not examined in this study.
