# Correction: Relationship between ultrasonographic findings and subscales of the Knee Injury and Osteoarthritis Outcome Score in patients with early knee osteoarthritis: a multicenter study

**DOI:** 10.1007/s10396-026-01641-9

**Published:** 2026-04-22

**Authors:** Yushin Mizuno, Yasushi Takata, Yosuke Shima, Kenichi Goshima, Kazunari Kuroda, Tomoyuki Kanayama, Yoshihiro Ishida, Naoki Takemoto, Manase Nishimura, Takuya Sengoku, Satoru Demura, Junsuke Nakase

**Affiliations:** 1 Department of Orthopaedic Surgery, Graduate School of Medical Sciences, Kanazawa University, 13-1 Takaramachi, Kanazawa, Ishikawa 920-8641 Japan; 2 Section of Rehabilitation, Kanazawa University Hospital, Kanazawa, Ishikawa Japan; 3 Department of Orthopaedic Surgery, KKR Hokuriku Hospital, Kanazawa, Ishikawa Japan; 4 Department of Orthopaedic Surgery and Joint Reconstructive Surgery, Kanazawa Munehiro Hospital, Kanazawa, Ishikawa Japan; 5 Department of Orthopaedic Surgery, Yawata Medical Center, Komatsu, Ishikawa Japan

**Correction: Journal of Medical Ultrasonics (2025) 52:139–148** 10.1007/s10396-024-01498-w

In the original version of this article, the conclusion of the abstract was published incorrectly. The old incorrect version and the corrected version of the conclusion are given below.

Incorrect version:

**Conclusion** Quality and difficulty of life of patients with early KOA may be influenced by synovial hyperplasia in the suprapatellar bursa, joint effusion, and MME values in the upright position. Among them, synovial hyperplasia of the suprapatellar bursa and amount of MME in the upright position were independently associated with the KOOS subscales.

Corrected version:

**Conclusion** Quality and difficulty of life of patients with early KOA may be influenced by synovial hyperplasia in the suprapatellar bursa, joint effusion, and MME values in the upright position. Among them, knee joint effusion and amount of MME in the upright position were independently associated with the KOOS subscales.

The original article has been corrected.

